# Effects of Biodanza^®^ SRT on Motor, Cognitive, and Behavioral Symptoms in Patients with Parkinson’s Disease: A Randomized Controlled Study

**DOI:** 10.3390/jpm14060588

**Published:** 2024-05-30

**Authors:** Carmine Vitale, Roberta Rosa, Valeria Agosti, Mattia Siciliano, Giuseppe Barra, Gianpaolo Maggi, Gabriella Santangelo

**Affiliations:** 1 Department of Medical, Motor and Wellbeing Sciences, University of Naples “Parthenope”, 80133 Naples, Italy; 2 ICS Maugeri Hermitage Napoli, 80145 Naples, Italy; gsp.barra@gmail.com; 3 Department of Primary Education Sciences, European University of Rome, 00163 Roma, Italy; roberta.rosa@unier.it; 4 Department of Human, Philosophical and Educational Sciences, University of Salerno, 84084 Fisciano, Italy; vaagosti@unisa.it; 5 Department of Advanced Medical and Surgical Sciences, University of Campania “Luigi Vanvitelli”, 80138 Naples, Italy; mattia.siciliano@unicampania.it; 6 Department of Psychology, University of Campania “Luigi Vanvitelli”, 81100 Caserta, Italy; gabriella.santangelo@unicampania.it

**Keywords:** Parkinson’s disease, Biodanza, complementary therapies, dance therapy, non-pharmacological intervention

## Abstract

Rolando Toro’s Biodanza (SRT) is a therapeutic strategy that uses movement, music, and emotions to induce integrative living experiences. The present study aims to explore the efficacy of a three-month SRT intervention on motor, cognitive, and behavioral symptoms in patients with Parkinson’s disease (PD). This study employed a randomized between-group design. Twenty-eight non-demented PD patients were enrolled in this study. Out of these, fourteen patients were assigned to the active treatment group using the Biodanza SRT system and fourteen to the untreated control group. The study group attended 2 h SRT classes once a week, completing twelve lessons in twelve weeks. All patients underwent: (i) a neurological examination to measure the severity of motor symptoms, balance, mobility, and risk of falls, and (ii) a neuropsychological battery to assess cognitive status, apathy, depressive symptomatology, and perceived quality of life (QoL), at study entry (T0) and at twelve weeks (T1, end of dance training). At T1, we observed a significant improvement in motor (i.e., severity of motor symptoms and balance) and cognitive parameters (i.e., working memory and delayed verbal memory) in all treated patients compared with the controls. Furthermore, a significant improvement in the social support dimension was found in all treated patients compared to the controls. A trend toward increased apathy was found in untreated patients at T1. The three-month Biodanza intervention significantly ameliorated the motor parameters of PD patients, with a parallel improvement in cognitive and QoL status. Hence, Biodanza intervention can, in the short term, represent a useful personalized medical intervention for the management of Parkinson’s disease.

## 1. Introduction

Parkinson’s disease (PD) is a progressive neurodegenerative disease associated with functional disability, social isolation, and reduced quality of life (QoL). Patients with PD present with motor disturbances, such as bradykinesia, tremor at rest, rigidity, and postural instability. These symptoms vary over time and limit the functional capacities of patients, who are no longer able to generate adequate motor responses to internal and external stress [1,2]. Axial symptoms and walking disorders also contribute to the impairment of patients’ functional autonomy and are associated with an increased risk of falling [3,4,5]. The severity of motor symptoms is related to disease progression, alterations in neurotransmitter balance, and drug treatment [6]. Alongside the cardinal symptoms of PD, a set of non-motor signs and symptoms, including cognitive impairment, autonomic dysfunction, sleep disturbances, and affective and behavioral disorders (i.e., apathy, depression, and anxiety), is equally disabling and affects QoL [7,8,9,10,11].

Dopamine replacement therapy remains the first-line treatment for amelioration of PD cardinal symptoms, but it rarely achieves optimal control [6]. Therefore, the management of PD patients requires an integrated multidisciplinary approach that includes non-pharmacological interventions (cognitive training, physical activity, and non-invasive brain stimulation) and complementary therapies aimed at achieving long-lasting symptomatic benefits and improving QoL. Several studies have demonstrated the short-term effectiveness of conventional physiotherapy in improving walking, postural stability, and patients’ QoL [12,13,14]. However, temporal continuity, adherence to physiotherapy programs, and conventional motor activity can vary over time [13,15]. The lack of adequate services, reduced expectations of efficacy, and low interest in the activities offered have been identified as factors contributing to failure of physical therapy among patients [16].

Engaging patients in stimulating activities that respect the needs and functional limitations of the disease can help overcome these barriers and encourage their regular participation. Other forms of physical activity, such as cycling, walking, postural exercise, hydrotherapy, and martial arts, have proven beneficial in terms of improving patients’ mobility and quality of life, in addition to traditional therapies [12,13,17,18,19,20,21,22].

A growing body of experimental evidence demonstrates that dance can be an effective form of exercise for PD patients by combining attentional stimuli with highly selective and specific motor tasks [15,23,24]. In addition to being a highly engaging and aggregating physical activity, dance seems to enhance body awareness and the perception of well-being, improving movement control through the use of sensory stimuli, cognitive strategies, creativity, and rhythm [25]. Although the use of dance therapy in PD is becoming popular, the contribution of some factors, such as external stimuli, music, the presence of a partner, and the instructions given by a dance teacher, remains unclear [18,23]. Furthermore, the long-term effects of dance therapy have not been confirmed in all studies [26]. In a recent meta-analysis, Cheng and collaborators [18] showed that the type, frequency, and duration of dance classes were associated with improvements in mental health. Compared with traditional rehabilitation techniques, dance therapy appears to be more effective in improving balance, strength, and movement strategies [27]. However, only a few studies have evaluated the effects of dance therapy on the cognitive and behavioral aspects of PD [28].

Biodanza is a psychophysical integration technique based on movement, music, and group interaction [29], and it has demonstrated beneficial effects in various clinical and non-clinical population therapeutic areas, from rheumatological and osteoarticular pathologies to neuropsychiatric diseases [30,31,32,33]. A preliminary study conducted on a small cohort of PD patients highlighted an improvement in motor parameters and body awareness, together with an increase in the perception of subjective well-being in the Biodanza treatment group [34]. The feasibility and effectiveness of Biodanza-based intervention have been demonstrated among elderly residents of nursing homes, with improvements in neuropsychiatric symptoms and overall well-being of individuals with Alzheimer’s disease [35].

Taking into account this background, we hypothesized that PD patients might benefit from a Biodanza SRT intervention in terms of improvements in motor and non-motor aspects of PD, including cognitive abilities, behavioral symptoms, and their perceived QoL. Therefore, in the present randomized controlled study, we evaluated the effects of Biodanza SRT on the motor, cognitive, and behavioral symptoms of PD patients. For these purposes, we enrolled 28 PD patients who were randomly assigned (1:1 ratio) to a 12-week active Biodanza treatment group or a control untreated group. Neurological, cognitive, and behavioral parameters of the two groups were assessed at baseline (T0) and at the end of treatment (T1) to reveal possible changes due to the active intervention.

## 2. Materials and Methods

### 2.1. Participants

Twenty-eight PD outpatients (twenty-two males and six females) were enrolled at the Movement Disorders Center of the “ICS Maugeri Hermitage Napoli” of Naples, Italy, using convenience sampling. Participants met the following inclusion criteria: (i) a diagnosis of idiopathic PD confirmed by a neurologist experienced in movement disorders based on clinical diagnostic criteria [36], (ii) preserved cognitive functioning defined according to age- and education-adjusted scores on the Mini Mental State Examination above the cut-off (MMSE > 23.8) [37], and (iii) absence of psychiatric disturbances. Furthermore, participants were excluded if they had concomitant medical causes that contraindicated participation in the treatment protocol and if they were participating in other individual or group motor activities in the period prior to enrollment and during the treatment period until the end of the treatment period. The selected patients signed an informed consent form before being included in the experimental protocol, which was conducted in accordance with the principles of the Declaration of Helsinki.

### 2.2. Evaluation Procedure

**Figure 1:**
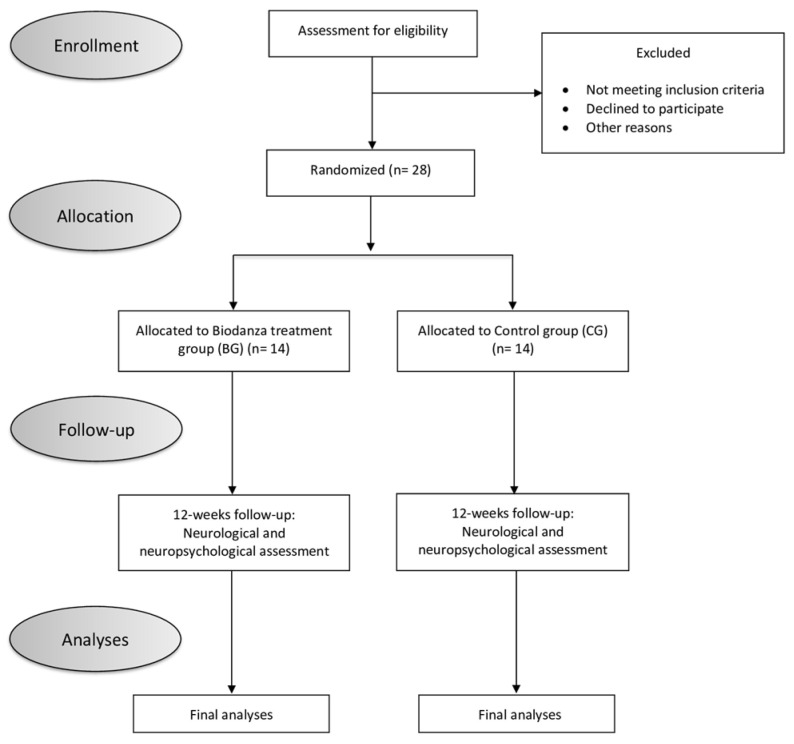
Flow diagram of the randomized controlled trial.

All patients underwent neurological and neuropsychological evaluations during the recruitment phase (T0) and at the end of the treatment (T1). The following clinical and demographic variables were recorded: age, sex, age at PD onset, disease duration, and the dominant clinical side. Treatment with concomitant drugs has also been reported in the drug history of the enrolled patients. The recruited patients were randomly assigned (1:1 ratio) to an active Biodanza treatment group (BG) or to a control group (CG) that did not participate in active treatment and/or motor activity for the following 12 weeks (Figure 1). The pharmacological therapy of the recruited patients remained stable until the end of treatment (T1). No additions or reductions to the dosing regimen recorded at the baseline visit were allowed during this period.

### 2.3. Neurological Evaluation

The clinical assessments were performed in a single morning session lasting approximately two hours, when patients were in the “ON” phase. Short breaks were made to prevent fatigue. The neurological examination included: (1) the motor section of the Unified Parkinson’s Disease Rating Scale (UPDRS-III) to measure the severity of motor symptoms in the” ON “phase, (2) the Hoehn and Yahr PD staging scale (H&Y), and (3) the Timed Up and Go scale (TUG, consists of measuring how many seconds it takes the patient to get up from the chair, walk a distance of 3 m, turn around, go back to the chair, and sit down again; normal time: between 7 and 10 s, high risk of falling: >20 s), for assessing the state of mobility and the risk of falls [38]. The Levodopa Equivalent Daily Dose (LEDD) was calculated for dopamine agonists + L-Dopa (total LEDD) [39].

### 2.4. Neuropsychological Evaluation

All participants underwent a neuropsychological assessment consisting of: (1) the Italian version of the Parkinson’s Disease Cognitive Rating Scale (PD-CRS), which explores subcortical functions (immediate and delayed verbal recall, sustained attention, working memory, clock drawing, and alternating verbal fluency) and cortical functions (naming and clock copy) [40,41], (2) the Self-Report Version of the Apathy Evaluation Scale (AES-S) [42] to assess apathy, (3) the Beck Depression Inventory-II (BDI-II) [43] to evaluate depressive symptoms, and (4) the Parkinson’s Disease Questionnaire [44], which measures quality of life and consists of 39 items (PDQ-39) evaluating 8 different domains (mobility, daily life activity, emotional well-being, stigma, social support, cognitive impairment, communication, and physical discomfort), where each domain is scored from 0 (good quality of life) to 100 (bad quality of life).

### 2.5. Biodanza SRT Intervention Protocol

Participants in the active treatment group followed the Biodanza SRT program, which comprised weekly meetings lasting 2 h for 12 weeks (3 months). Each Biodanza session was structured in two phases: (1) The first phase about sharing, verbalization, and restitution of experiences, served as a theoretical preparation for motor activity, as a stimulus to verbal sharing through the use of the excited word (with which the patient expresses their authentic feeling), and deepening of mutual knowledge (duration: approximately 45–50 min). (2) The second motor/experiential/“vivenciale” phase involved the proposition of dances/exercises, illustrated by a therapist, supported and favored by music and accompanied by an expressive example and precise existential motivations about the experience that one is invited to live (duration: about 60–70 min).

The dances/exercises were structured with respect to the original theoretical model and calibrated in relation to the type of user, following a physiological curve, which included: a first activating phase (intensification of self-awareness) and a second regression phase (amplification of the self-consciousness). The dances/exercises pursue specific objectives: (1) motor integration, (2) affective-motor integration, (3) affective communication, (4) communion, and (5) expression of genetic potentials. They can be practiced individually (to develop self-perception), in pairs (to develop the perception of feedback, active listening, respect, and care), in small groups, and/or with the whole group (to experience healthy relationships through affective communication with oneself, others and the surrounding world). The dances/exercises scheme proposed for the cohort of enrolled Parkinsonian patients had the purpose of (1) creating new movement patterns that integrate the emotional and motor components of the individual, and (2) generating adaptive responses aimed at enhancing communication and self-esteem (Table 1).

### 2.6. Statistical Analysis

Differences between the baseline (T0) characteristics of the two groups were assessed using the Mann–Whitney U and Pearson Chi-squared (ꭓ^2^) tests. To investigate differences in motor, cognitive, and behavioral variables after the Biodanza SRT program, we carried out several 2 × 2 mixed-design ANOVA, with time (i.e., T0 and T1) as the within-subject factor and group (i.e., CG and BG) as the between-subject factor. Bonferroni-corrected post hoc comparisons were performed for significant results. Statistical analysis was performed with SPSS version 26 software (SPSS Inc., Chicago, IL, USA).

## 3. Results

### 3.1. Baseline Groups’ Comparison

The two groups were matched for age, clinical variables (i.e., disease duration, disease staging, severity of motor symptoms, and total LEDD), and cognitive variables (i.e., PDCRS scores) at baseline (T0) (Supplementary Materials Table S1). No significant differences emerged in behavioral variables (i.e., AES, BDI-II, and PDQ-39) between the two groups (Supplementary Materials Table S1).

### 3.2. Effect of Biodanza SRT Program on Motor Variables

**Figure 2:**
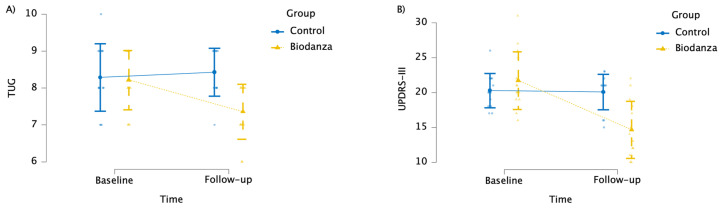
Graph showing the effect of the Biodanza SRT program on (**A**) TUG and (**B**) UPDRS-III variables.

Analysis of TUG using mixed ANOVA revealed that the interaction between time and group was significant (*F*_(1, 26)_ = 8.493; *p* = 0.007; $\eta_{p}^{2}$ = 0.246) (Supplementary Materials Table S2). Bonferroni-corrected post hoc comparisons showed that the BG group reported better mobility (TUG) than the CG at T1 (corrected-*p* < 0.001); moreover, TUG scores decreased at T1 (mean = 7.36; SD = 0.74) compared to T0 (mean  =  8.21; SD = 0.80) within the BG (corrected-*p* = 0.002) but not within the CG (corrected-*p* = 0.561) (Figure 2A).

Moreover, analysis of the UPDRS-III scores showed a significant interaction between time and group (*F*_(1, 26)_ = 28.594; *p* < 0.001; $\eta_{p}^{2}$ = 0.524) (Supplementary Materials Table S2). In detail, Bonferroni-corrected post hoc comparisons revealed that the BG presented less severe motor symptoms (UPDRS-III) than the CG at T1 (corrected-*p* < 0.001); in addition, the UPDRS-III score improved at T1 (mean = 14.64; SD = 4.09) compared to baseline (mean = 21.71; SD = 4.14) within the BG (corrected-*p* < 0.001), whereas it remained stable within the CG (corrected-*p* = 0.815) (Figure 2B).

### 3.3. Effect of Biodanza SRT Program on Cognitive Variables

**Figure 3:**
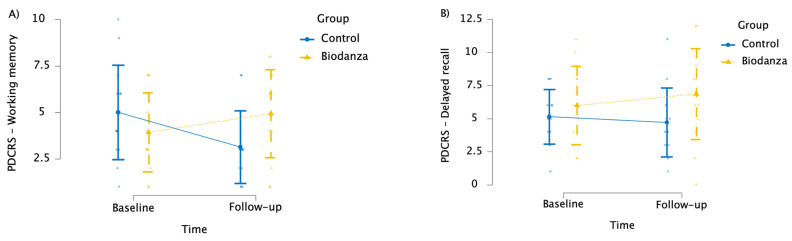
Graph showing the effect of the Biodanza SRT program on the (**A**) PDCRS—working memory and (**B**) PDCRS—delayed recall subtests.

We found a significant interaction between time and group on the working memory subtest of the PDCRS (*F*_(1, 26)_ = 32.500; *p* < 0.001; $\eta_{p}^{2}$ = 0.556) (Supplementary Materials Table S3). In detail, Bonferroni-corrected post hoc comparisons showed that the BG reported better working memory abilities compared to the CG at T1 (corrected-*p* = 0.039); in addition, working memory abilities improved at T1 (mean = 4.93; SD=2.37) compared to T0 (mean = 3.93; SD = 2.13) within the BG (corrected-*p* = 0.009), whereas they worsened (T0: mean = 5.00; SD = 2.54; T1: mean = 3.14; SD = 1.96) within the CG (corrected-*p* < 0.001) (Figure 3A).

We observed a significant interaction between time and group on the delayed verbal memory recall subtest of the PDCRS (*F*_(1, 26)_ = 5.456; *p* = 0.027; $\eta_{p}^{2}$ = 0.173) (Supplementary Materials Table S3). More specifically, Bonferroni-corrected post hoc comparisons showed that verbal memory abilities improved at T1 (mean = 6.86; SD = 3.44) compared to T0 (mean = 6.00; SD = 2.96) within the BG (corrected-*p* = 0.037) but not within the CG (corrected-*p* = 0.281) (Figure 3B).

A significant interaction emerged between time and group on the alternating fluency subtest of the PDCRS (*F*_(1, 26)_ = 6.251; *p* = 0.019; $\eta_{p}^{2}$ = 0.194) (Supplementary Materials Table S3). More specifically, Bonferroni-corrected post hoc comparisons revealed that alternating fluency scores worsened at T1 (mean = 4.93; SD = 1.98) compared to T0 (mean = 7.36; SD = 5.61) within the CG (corrected-*p* = 0.010), whereas they remained stable within the BG (corrected-*p* = 0.466).

No significant effect of treatment was observed on the PDCRS total, cortical, and subcortical scores or on the other subtests (Supplementary Materials Table S3).

### 3.4. Effect of Biodanza SRT Program on Behavioral Variables

**Figure 4:**
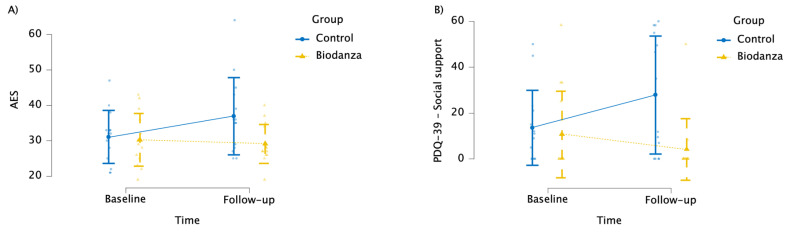
Graph showing the effect of the Biodanza SRT program on the (**A**) Apathy Evaluation Scale and the (**B**) PDQ-39—social support.

We observed a trend toward significance on the interaction between time and group on AES (*F*_(1, 26)_ = 3.371; *p* = 0.078; $\eta_{p}^{2}$ = 0.115) (Supplementary Materials Table S4). Bonferroni-corrected post hoc comparisons showed that the CG reported more severe apathy symptoms than the BG at T1 (corrected-*p* = 0.025); moreover, the apathy score increased within the CG (corrected-*p*=0.039), whereas it remained stable within the BG (corrected-*p* = 0.675) (Figure 4A). Conversely, no significant effect of treatment was observed on BDI scores (*F*_(1, 26)_ = 0.009; *p* = 0.924; $\eta_{p}^{2}$ = 0.000).

As for PDQ-39 dimensions, a significant interaction emerged between time and group on the social support dimension (*F*_(1, 26)_ = 6.406; *p* = 0.018; $\eta_{p}^{2}$ = 0.198) (Supplementary Materials Table S4). In detail, Bonferroni-corrected post hoc comparisons revealed that the BG perceived more social support (mean = 4.17; SD = 13.38) than the CG (mean = 27.92; SD = 25.76) at T1 (corrected-*p* = 0.005) but not at T0 (corrected-*p* = 0.665); moreover, social support perception worsened at T1 (mean = 27.92; SD = 25.76) compared to T0 (mean = 13.64; SD = 16.37) within the CG (corrected-*p* = 0.021), whereas it remained stable within the BG (corrected-*p* = 0.271) (Figure 4B). In the same way, the interaction between time and group on the physical discomfort dimension was significant (*F*_(1, 26)_ = 4.423; *p* = 0.045; $\eta_{p}^{2}$ = 0.145) (Supplementary Materials Table S4). In detail, Bonferroni-corrected post hoc comparisons showed that the CG perceived more physical discomfort (mean = 59.80; SD = 20.66) than the BG (mean = 39.29; SD = 22.27) at T1 (corrected-*p* = 0.018) but not at T0 (corrected-*p* = 0.443); moreover, physical discomfort perception worsened at T1 (mean = 59.80; SD = 20.66) compared to T0 (mean = 29.51; SD = 29.15) within the CG (corrected-*p* = 0.003), whereas it remained stable within the BG (corrected-*p* = 0.802). No significant effects of treatment were observed on the other PDQ-39 dimensions (Supplementary Materials Table S4).

## 4. Discussion

The present study aimed to evaluate the effectiveness of the Biodanza system on motor, cognitive, and behavioral symptoms in patients with PD. We found that patients showed improvements in terms of severity of motor symptoms and postural stability after the Biodanza program. Furthermore, patients in the Biodanza group showed better cognitive performance in tests evaluating verbal memory recall and working memory. Finally, after the Biodanza program, patients experienced a better QoL in terms of greater perceived social support compared to patients in the control group, who reported a trend of greater apathy and more severe physical discomfort. No side effects were observed at the end of active Biodanza treatment.

Regarding motor variables, our results showed a significant reduction in the UPDRS-III score in the Biodanza treatment group at the end of the intervention. The sequences and steps of exercises proposed by the Biodanza system improve motor function by enhancing muscle lengthening, ensuring the optimal maintenance of balance, and promoting greater fluidity of movement. Furthermore, the support of rhythm and music also stimulates cognitive functions, inducing the planning and execution of imagined movements, and enhancing the memory of repeated actions and awareness of one’s body. Therefore, it is possible to hypothesize that the combined stimulation of motor and cognitive functions can also produce synergistic activation of the subcortical circuits of the basal ganglia and other brain areas involved in the planning and execution of motor tasks, as demonstrated by the results of previous studies. Using positron emission tomography (PET), Brown and Lawrence demonstrated that blood flow to the motor areas and cerebellum increases when dance steps are performed [45]. Calvo-Merino et al. [46] and Cross et al. [47] hypothesized selective activation of the brain areas responsible for movement planning in response to dance movements. In line with these hypotheses, Sacco and collaborators demonstrated that tango lessons stimulated the activation of the premotor and supplementary motor areas [48].

Furthermore, we observed a significant reduction in TUG scores only within the Biodanza group compared to the baseline assessment, with repercussions in terms of greater postural stability and a reduction in the risk of falling. The improvements recorded in TUG times at the end of active treatment have been reported also in previous studies that adopted other types of dance therapy, despite this measure seems to be less sensitive to the intervention effects than the other measures of functional mobility [28,49,50]. Hackney and Earhart also demonstrated that complex tango steps produced improvement effects in terms of balance and postural stability [51]. As for tango, the sequence of exercises and steps developed for our study included rhythmic combinations and repetitions of antero-posterior and lateral steps, aimed at achieving greater stability and awareness of the sense of position in space, as well as generating adaptive responses to the changes in stresses in the internal and external environments. These improvements in motor scores and global mobility achieved at the end of treatment could be the result of a combined effect on the motor and non-motor symptoms of PD, affecting patients’ overall mobility and balance.

The group of patients who followed the Biodanza protocol also showed a significant improvement in cognitive abilities, specifically in working memory and long-term verbal memory, compared to the baseline assessment, while the control group showed a decrease in cognitive flexibility. Kalyani and colleagues demonstrated that dance is effective in improving cognitive function [28] by promoting the activation of brain areas that normally show reduced activity in PD patients [52]. A PET study also demonstrated that dancing stimulates interacting neural networks in several cortical, subcortical, and cerebellar regions [53]. The practice of dance requires a concentration of attentional resources on the music and external signals while imagining the next movement and proceeds through the learning of consecutive gestures from the simplest to the most complex. In other words, motor imagery activates the motor circuits of the basal ganglia and frontal lobe, influencing memory functions and the selection and execution of gestures [54]. Dance is a physical activity that requires harmonious integration of cognitive functions, such as perception, executive functions, memory, and motor skills [55]. The results of this study suggest an improving effect of Biodanza on memory functions and the ability to strategically recall some aspects of episodic memory mediated by the prefrontal cortex [56]. This result could be explained by the fact that there are at least two sources of cognitive stimulation derived from the act of dancing: the musical element and the complex physical and motor skills required to perform the steps [57]. Furthermore, listening to music produces the selective activation of various sensorimotor, cognitive, and emotional circuits of the brain [58], especially of the temporal and frontal areas, with consequent enhancement of verbal functions memory [59]. The retrieval of information from verbal memory is also trained by the fact that participants should pay attention and concentrate attentional resources on the instructions provided by the therapist in each session, since they must retrieve instructions from previous sessions and discuss them in groups before starting a new session. This mechanism produces stimulation and “training” of the ability to keep verbal information active and recall it at the right time.

As for the behavioral variables, at the end of the treatment, the experimental group did not show significant improvements in scores on the apathy and depression scales, whereas the control group showed a slight increase in apathy symptoms. Therefore, the maintenance of stable scores on the apathy rating scale was strongly influenced by the intervention, a result that further supports the peculiar characteristics of the Biodanza system. Indeed, through the combined use of music and movement, Biodanza induces pleasant sensations. Whether patients dance in pairs or small groups, the resulting interactions strengthen the feeling of unity and sharing. Dance can generate pleasure through mutual understanding and shared emotions between participants experiencing the same health problems, improving mood, relieving anxiety, and increasing motivation [60,61,62]. The resulting sensation of pleasure and well-being may act on motivation by modulating the flow of information between the basal ganglia and cortico–subcortical reward circuits [63]. However, this aspect should be further investigated, because apathy is one of the most frequent non-motor symptoms and has been associated with poor awareness [64], more severe cognitive impairment [65,66], other neuropsychiatric disorders [67], and poor QoL in PD.

Finally, the results of our study also demonstrated an improvement in the quality of life of treated patients, similar to that reported in previous studies (tango, tai-chi, and ballroom dances) [68,69,70]. This improvement was evident in the social support and physical discomfort dimensions of the patients who participated in the Biodanza sessions. This result is in line with those of other studies on the positive effects of group dance on social and interpersonal skills. Dance is an aggregating activity that promotes social relationships in older people [71,72], which has been associated with an improvement in cognitive performance in longitudinal studies [73]. The intrinsic social factor in dance could also be the basis of improved cognitive function, in particular, a better ability to strategically recall information and greater motivation and interest in general. Finally, patients in the control group reported an increase in physical discomfort, whereas this was not observed in patients who participated. This finding is in line with previous evidence, revealing the beneficial effect of dance in producing a shift from painful to pleasant bodily sensations and relieving pain in PD patients [74] and other clinical groups [75].

The small sample size, recruited using convenience sampling, represents the main limitation of the present study, which reduces the generalizability of our findings. However, it should be noted that functional limitations and logistical difficulties strictly related to the disease, as well as the reduced expectation for efficacy of patients and caregivers, are often challenging for active enrollment in this type of clinical trial. Furthermore, we must emphasize that some baseline differences regarding the type and amount of dopaminergic replacement therapy might represent a source of bias in our results. However, we tried to control for this confounding variable by avoiding any changes to the therapy recorded at baseline. Finally, we did not evaluate the long-term effects of the treatment. Although an increasing number of experimental trials have demonstrated the beneficial effects of dance in PD patients, the long-term effects of dance therapy have not been confirmed in all studies. Future studies should use larger samples to improve the generalizability of the results and evaluate the long-term effects of the Biodanza system while controlling for the possible effects of medication.

## 5. Conclusions

Although these results may be considered preliminary evidence, Biodanza has proven to be an effective form of complementary therapy, at least in the short term, capable of significant improvements in both the motor and non-motor aspects of PD by incorporating physical elements of exercise with psychosocial therapeutic components. In addition to the benefits of balance and motor aspects in general, PD patients who underwent the Biodanza SRT program showed enhanced cognitive functions (i.e., working memory and verbal memory), as well as the perception of social support. Although patients in the active treatment group did not show improvements in apathy or depression scores, the control group reported an increase in apathy severity. These results indirectly suggest that the worsening of some non-motor symptoms could be observable even over a period of three months and, therefore, underline the need to extend the observation period over longer periods to obtain a reliable estimate of the progression of the disease in relation to the motor and non-motor aspects of PD.

## Figures and Tables

**Table 1:** Structure and contents of the Biodanza dances/exercises in relation to motor and non-motor symptoms of PD.

PD Symptoms	Dances/Exercises Biodanza SRT
Hypo/akinesia (mainly when walking)	Rhythmic walking exercises. Synergistic walking.
Tremor	Dances with emotional motivation.
Rigidity	Fluidity Exercises. Free and fluid dances.
Difficulty initiating voluntary movements	Creative dances and “giving and receiving the flower”. Slow dances with expressive variations.
Difficulty initiating voluntary movements	Coordination dances with another person. Dances of Eutony.
Difficulty performing complex, fast, and alternating motor sequences	Rhythmic dances with variations. Samba. “Jazz” dances (Dixieland).
Postural alterations (antero-lateral flexion of the trunk)	Integration exercises of the cephalic, pectoral, and pelvic centers. Exercises of affective-motor integration.
Speech alterations	Chorus of “Divine Reed”. Singing of one’s name with expressive variations.
Micrograph	Proximity–distance coordination exercises.
Orthostatic hypotension and other parasympathetic symptoms	Exercises of the ergotropic (adrenergic) series.
Depression	Euphoric dances. Communication dances. Dance of the seed.
Self-doubt	Exercises of making contact with one’s own strength. Yang dance. Walk with determination
Sense of inferiority	Creative dances in the center of the circle. Encounter dances. Dances of rebirth.
Inexpressiveness and communication difficulties	Dances of expression of emotions. Dances of Love.

## Data Availability

The original contributions presented in the study are included in the article, further inquiries can be directed to the corresponding authors.

## Supplementary Materials

The following supporting information can be downloaded at: https://www.mdpi.com/article/10.3390/jpm14060588/s1, Table S1. Demographical and clinical characteristics of the Biodanza and Control groups at baseline assessment (T0). Table S2. Descriptive statistics and mixed ANOVA results on motor variables. Table S3. Descriptive statistics and mixed ANOVA results on cognitive variables. Table S4. Descriptive statistics and mixed ANOVA results on behavioral variables.
