# Supplementary material for: Effects of Biodanza® SRT on Motor, Cognitive, and Behavioral Symptoms in Patients with Parkinson’s Disease: A Randomized Controlled Study
Source: J Pers Med. 2024 May 30;14(6):588. doi: 10.3390/jpm14060588 (PMC11204495; doi:10.3390/jpm14060588)
Supplement: Supplementary file 1 [file jpm-14-00588-s001.zip › jpm-3034526-supplementary.pdf]

Supplementary Material Table S1. Demographical and clinical characteristics of the Biodanza and Control groups at baseline assessment (T0).

	Control Group (N=14)	Biodanza Group (N=14)	U	*p*
**Age**	62.9 ± 4.7	64.1 ± 7.9	83.000	0.511
**Sex**	M= 10 (71.43%)<br>F= 4 (28.57%)	M= 12 (85.71%)<br>F= 2 (14.29%)	0.848	0.357
**Disease Duration/ys**	6.5 ± 3.5	5.5 ± 2.8	76.500	0.329
**UPDRS-III**	20.3 ± 2.4	21.7 ± 4.3	84.000	0.541
**TUG**	8.3 ± 0.9	8.2 ± 0.8	95.000	0.910
**H&Y**	1.7 ± 0.6	2.0 ± 0.0	54.000	0.222
**Total-LEDD/mg**	705 ± 121.2	603.5 ± 230.7	73.500	0.265
**PDCRS total**	76.1 ± 23.9	74.0 ± 15.7	93.000	0.839
**PDCRS cortical**	23.3 ± 5.2	24.9 ± 4.0	84.500	0.541
**PDCRS subcortical**	52.8 ± 19.4	49.1 ± 12.1	79.000	0.401
**PDCRS – Immediate recall – verbal memory**	7.4 ± 1.5	7.9 ± 2.1	91.500	0.769
**PDCRS - Naming**	14.9 ± 3.8	16.2 ± 2.8	82.000	0.482
**PDCRS - Attention**	7.4 ± 2.4	8.4 ± 1.2	76.000	0.329
**PDCRS – Working memory**	5.0 ± 2.5	3.9 ± 2.1	74.500	0.285
**PDCRS – Clock-drawing**	8.4 ± 1.8	7.8 ± 2.1	77.000	0.352
**PDCRS – Clock-copy**	8.4 ± 1.9	8.6 ± 1.4	92.500	0.804
**PDCRS – Delayed recall - verbal memory**	5.1 ± 2.1	6.0 ± 3.0	82.500	0.482
**PDCRS – Alternating fluency**	7.4 ± 5.7	4.2 ± 2.1	68.000	0.178
**PDCRS – Action fluency**	12.1 ± 7.1	10.0 ± 4.1	80.000	0.427
**AES**	31.1 ± 7.4	30.3 ± 7.4	89.500	0.701
**BDI-II**	8.5 ± 8.4	12.1 ± 10.1	60.500	0.085
**PDQ-39 – Mobility**	34.7 ± 20.2	20.3 ± 14.5	57.000	0.062
**PDQ-39 – Daily activities**	27.6 ± 15.1	25.8 ± 17.3	97.500	0.982
**PDQ-39 – Psychological Wellbeing**	25.7 ± 16.9	25.2 ± 21.2	90.000	0.734
**PDQ-39 – Stigma**	19.8 ± 26.7	12.5 ± 5.6	90.000	0.734
**PDQ-39 – Social support**	13.6 ± 16.3	15.4 ± 19.7	75.000	0.306
**PDQ-39 – Cognitive impairment**	21.0 ± 22.5	27.2 ± 19.8	69.000	0.194
**PDQ-39 – Communication**	11.3 ± 14.5	16.1 ± 25.6	95.500	0.910
**PDQ-39 – Physical discomfort**	29.5 ± 29.1	35.1 ± 22.4	71.000	0.227

UPDRS-III, Unified Parkinson's Disease Rating Scale; TUG, Time up and go scale; H&Y, Hoehn and Yahr Scale; LEDD, L-dopa daily equivalent dose; PDCRS, Parkinson's Disease Cognitive Rating Scale; AES, Apathy Evaluation Scale; BDI-II, Beck Depression Inventory-II; PDQ-39, 39-item Parkinson's disease Questionnaire

Supplementary Material Table S2. Descriptive statistics and mixed ANOVA results on motor variables.

Variables	Intervention				ANOVA		
	Biodanza		Control		Within	Between	Interaction
	T0	T1	T0	T1			
TUG					[$F_{(1, 26)}$= 4.333*; η_p^2= 0.143]	[$F_{(1, 26)}$= 5.622*; η_p^2= 0.178]	[$F_{(1, 26)}$= 8.493**; η_p^2= 0.246]
Mean	8.21	7.36	8.29	8.43			
SD	0.80	0.74	0.91	0.65			
UPDRS-III					[$F_{(1, 26)}$= 32.280***; η_p^2= 0.554]	[$F_{(1, 26)}$= 3.206; η_p^2= 0.110]	[$F_{(1, 26)}$= 28.594***; η_p^2= 0.524]
Mean	21.71	14.64	20.29	20.07			
SD	4.14	4.09	2.46	2.56			

*$p<0.05$, **$p>0.01$, ***$p<0.001$

UPDRS-III, Unified Parkinson's Disease Rating Scale; TUG, Time up and go scale.

Supplementary Material Table S3. Descriptive statistics and mixed ANOVA results on cognitive variables.

Variables	Intervention				ANOVA		
	Biodanza		Control		Within	Between	Interaction
	T0	T1	T0	T1			
PDCRS total					[$F_{(1, 26)}$= 0.075; η_p^2= 0.003]	[$F_{(1, 26)}$= 0.043; η_p^2= 0.002]	[$F_{(1, 26)}$= 0.175; η_p^2= 0.007]
Mean	73.00	76.07	76.07	75.43			
SD	15.66	18.49	24.01	18.77			
PDCRS cortical					[$F_{(1, 26)}$= 6.612*; η_p^2= 0.203]	[$F_{(1, 26)}$= 0.021; η_p^2= 0.001]	[$F_{(1, 26)}$= 3.044; η_p^2= 0.105]
Mean	24.86	25.50	23.29	26.64			
SD	4.05	3.90	5.27	4.34			
PDCRS subcortical					[$F_{(1, 26)}$= 0.264; η_p^2= 0.010]	[$F_{(1, 26)}$= 0.749; η_p^2= 0.028]	[$F_{(1, 26)}$= 0.015; η_p^2= 0.001]
Mean	48.14	50.57	52.86	54.36			
SD	12.07	15.22	19.43	18.12			
Immediate recall					[$F_{(1, 26)}$= 2.572; η_p^2= 0.090]	[$F_{(1, 26)}$= 0.027; η_p^2= 0.001]	[$F_{(1, 26)}$= 1.077; η_p^2= 0.040]
Mean	7.86	8.07	7.36	8.36			
SD	2.11	2.27	1.50	1.98			
Naming					[$F_{(1, 26)}$= 3.048; η_p^2= 0.105]	[$F_{(1, 26)}$= 0.937; η_p^2= 0.035]	[$F_{(1, 26)}$= 0.173; η_p^2= 0.007]
Mean	16.21	16.79	14.93	15.86			
SD	2.83	2.81	3.83	3.35			
Attention					[$F_{(1, 26)}$= 2.991; η_p^2= 0.103]	[$F_{(1, 26)}$= 6.779*; η_p^2= 0.207]	[$F_{(1, 26)}$= 0.377; η_p^2= 0.014]
Mean	8.36	7.64	7.36	5.86			
SD	1.22	2.50	2.34	2.51			
Working Memory					[$F_{(1, 26)}$= 2.925; η_p^2= 0.101]	[$F_{(1, 26)}$= 0.191; η_p^2= 0.007]	[$F_{(1, 26)}$= 32.500***; η_p^2= 0.556]
Mean	3.93	4.93	5.00	3.14			
SD	2.13	2.37	2.54	1.96			
Clock-drawing					[$F_{(1, 26)}$= 0.056; η_p^2= 0.002]	[$F_{(1, 26)}$= 0.432; η_p^2= 0.016]	[$F_{(1, 26)}$= 0.306; η_p^2= 0.012]
Mean	7.79	8.14	8.43	8.29			
SD	2.08	1.87	1.83	2.13			
Clock-copy					[$F_{(1, 26)}$= 0.868; η_p^2= 0.032]	[$F_{(1, 26)}$= 0.022; η_p^2= 0.001]	[$F_{(1, 26)}$= 0.489; η_p^2= 0.018]
Mean	8.64	8.71	8.36	8.86			
SD	1.39	1.44	1.91	1.23			
Delayed recall					[$F_{(1, 26)}$= 0.606; η_p^2= 0.023]	[$F_{(1, 26)}$= 2.129; η_p^2= 0.076]	[$F_{(1, 26)}$= 5.456*; η_p^2= 0.173]
Mean	6.00	6.86	5.14	4.71			
SD	2.96	3.44	2.07	2.61			
Alternating fluency					[$F_{(1, 26)}$= 2.113; η_p^2= 0.075]	[$F_{(1, 26)}$= 2.040; η_p^2= 0.073]	[$F_{(1, 26)}$= 6.251*; η_p^2= 0.194]
Mean	4.21	4.86	7.36	4.93			
SD	2.12	2.48	5.61	1.98			
Action fluency					[$F_{(1, 26)}$= 0.522; η_p^2= 0.020]	[$F_{(1, 26)}$= 1.642; η_p^2= 0.059]	[$F_{(1, 26)}$= 0.374; η_p^2= 0.014]
Mean	10.00	10.07	12.07	12.93			
SD	4.08	5.47	7.12	4.23			

**p<0.05, **p>0.01, ***p<0.001*

PDCRS, Parkinson's Disease Cognitive Rating Scale

Supplementary Material Table S4. Descriptive statistics and mixed ANOVA results on behavioral variables.

Variables	Intervention				ANOVA		
	Biodanza		Control		Within	Between	Interaction
	T0	T1	T0	T1			
AES					[$F_{(1, 26)}$= 1.529; η_p^2= 0.056]	[$F_{(1, 26)}$= 3.252; η_p^2= 0.111]	[$F_{(1, 26)}$= 3.371; η_p^2= 0.115]
Mean	30.29	29.14	31.07	36.93			
SD	7.40	5.50	7.48	10.91			
BDI-II					[$F_{(1, 26)}$= 0.999; η_p^2= 0.037]	[$F_{(1, 26)}$= 1.603; η_p^2= 0.058]	[$F_{(1, 26)}$= 0.009; η_p^2= 0.000]
Mean	12.14	9.71	8.57	6.57			
SD	10.07	9.01	8.38	9.03			
PDQ-39 Mobility					[$F_{(1, 26)}$= 0.010; η_p^2= 0.000]	[$F_{(1, 26)}$= 4.947*; η_p^2= 0.160]	[$F_{(1, 26)}$= 0.001; η_p^2= 0.000]
Mean	20.53	20.36	34.71	34.32			
SD	16.12	14.51	20.20	21.55			
PDQ-39 Daily Activities					[$F_{(1, 26)}$= 1.752; η_p^2= 0.063]	[$F_{(1, 26)}$= 1.203; η_p^2= 0.044]	[$F_{(1, 26)}$= 1.376; η_p^2= 0.050]
Mean	25.89	26.49	27.63	37.51			
SD	17.39	20.84	15.11	20.51			
PDQ-39 Psychological Wellbeing					[$F_{(1, 26)}$= 0.658; η_p^2= 0.025]	[$F_{(1, 26)}$= 0.277; η_p^2= 0.011]	[$F_{(1, 26)}$= 0.398; η_p^2= 0.015]
Mean	25.30	19.64	25.71	25.01			
SD	21.21	13.21	16.91	19.07			
PDQ-39 Stigma					[$F_{(1, 26)}$= 0.300; η_p^2= 0.011]	[$F_{(1, 26)}$= 5.345*; η_p^2= 0.171]	[$F_{(1, 26)}$= 0.469; η_p^2= 0.018]
Mean	12.50	11.61	19.83	27.83			
SD	15.70	12.70	26.70	28.05			
PDQ-39 Social support					[$F_{(1, 26)}$= 0.883; η_p^2= 0.033]	[$F_{(1, 26)}$= 5.016*; η_p^2= 0.162]	[$F_{(1, 26)}$= 6.406*; η_p^2= 0.198]
Mean	10.71	4.17	13.64	27.92			
SD	18.90	13.38	16.37	25.76			
PDQ-39 Cognitive impairment					[$F_{(1, 26)}$= 5.639*; η_p^2= 0.178]	[$F_{(1, 26)}$= 0.005; η_p^2= 0.000]	[$F_{(1, 26)}$= 3.257; η_p^2= 0.111]
Mean	27.23	29.02	21.01	34.11			
SD	19.86	22.94	22.58	22.49			
PDQ-39 Communication					[$F_{(1, 26)}$= 1.773; η_p^2= 0.064]	[$F_{(1, 26)}$= 0.552; η_p^2= 0.021]	[$F_{(1, 26)}$= 2.032; η_p^2= 0.072]
Mean	16.07	15.48	11.30	28.73			
SD	25.62	15.28	14.54	30.52			
PDQ-39 Physical discomfort					[$F_{(1, 26)}$= 6.061*; η_p^2= 0.189]	[$F_{(1, 26)}$= 1.264; η_p^2= 0.046]	[$F_{(1, 26)}$= 4.423*; η_p^2= 0.145]
Mean	36.90	39.28	29.51	59.80			
SD	20.34	22.27	29.15	20.66			

**p<0.05, **p>0.01, ***p<0.001*

AES, Apathy Evaluation Scale; BDI-II, Beck Depression Inventory-II; PDQ-39, 39-item Parkinson's disease Questionnaire.
